# Empagliflozin reduces the senescence of cardiac stromal cells and improves cardiac function in a murine model of diabetes

**DOI:** 10.1111/jcmm.15699

**Published:** 2020-09-17

**Authors:** Rosalinda Madonna, Vanessa Doria, Ilaria Minnucci, Angela Pucci, Donato Sante Pierdomenico, Raffaele De Caterina

**Affiliations:** ^1^ Institute of Cardiology University of Pisa Pisa Italy; ^2^ Center of Aging Sciences and Translational Medicine – CESI‐Met "G. D'Annunzio" University, Chieti‐Pescara Chieti Italy; ^3^ Histopathology Department Pisa University Hospital Pisa Italy

**Keywords:** cardiac function, cardiac stromal cells, diabetes, empagliflozin, glucose, senescence

## Abstract

The sodium‐glucose cotransporter 2 (SGLT2) inhibitor empagliflozin reduces heart failure in diabetes, but underlying mechanisms remain elusive. We hypothesized that empagliflozin could counteract the senescence of cardiac stromal cells (CSC), the action of which limits cardiac damage and cardiac fibrosis in diabetic‐like conditions in vitro and in vivo. CSC were isolated from murine heart biopsies (n = 5) through cardiosphere (CSp) formation and incubated for 3 or 48 hours with 5.5 mmol/L normal glucose (NG), high glucose (12‐5 and 30.5 mmol/L, HG) or a hyperosmolar control of mannitol (HM) in the presence or absence of empagliflozin 100 nmol/L. The senescent CSC status was verified by β‐gal staining and expression of the pro‐survival marker Akt (pAkt) and the pro‐inflammatory marker p38 (p‐P38). The cardiac effects of empagliflozin were also studied in vivo by echocardiography and by histology in a murine model of streptozotocin (STZ)‐induced diabetes. Compared to NG, incubations with HG and HM significantly reduced the number of CSps, increased the β‐gal‐positive CSC and P‐p38, while decreasing pAkt, all reversed by empagliflozin (*P* < .01). Empagliflozin also reversed cardiac dysfunction, cardiac fibrosis and cell senescence in mice with (STZ)‐induced diabetes (*P* < .01). Empagliflozin counteracts the pro‐senescence effect of HG and of hyperosmolar stress on CSC, and improves cardiac function via decreasing cardiac fibrosis and senescence in diabetic mice, possibly through SGLT2 off‐target effects. These effects may explain empagliflozin unexpected benefits on cardiac function in diabetic patients.

## 1 INTRODUCTION

Heart failure (HF), with or without ischaemic heart disease, remains the leading causes of death worldwide.^1^ This can be attributed at least in part to the inability of the heart to regenerate damaged tissue.^2^ As a result, the lost myocardium is replaced with fibrosis, initiating a series of subsequent events that lead to adverse ventricular remodelling and organ failure.^3^ Cardiac stromal cells (CSC) are a heterogeneous population of non‐cardiomyocyte CD45‐, CD34‐, CD31‐ and CD105+ cells including fibroblasts, resident in the heart with supportive and paracrine functions. The crosstalk between cardiomyocytes and CSC plays a fundamental role for the repair processes after cardiac damage, through the release of growth factors, pro‐angiogenic factors and the regulation of cardiac metabolism.^4^, ^5^, ^6^ Over and above normal cellular ageing, exposure to cardiovascular risk factors accelerates the senescence of cardiomyocytes and CSC, hampering their cardioprotective and repairing functions, and ultimately leading to HF.^7^ One important condition that accelerates CSC senescence is diabetes.^7^ Diabetes features a state of accelerated ageing, as several fundamental age‐related changes occur at the cellular level in various tissues and organs (especially the blood vessels and the heart).^8^, ^9^ The crosstalk between CSC and cardiomyocytes can be hindered by the senescence of CSC due to metabolic and biophysical insults, such as in the presence of high glucose (HG) and related hyperosmolar stress, which can compromise cardiac function, promoting HF.^10^ In contrast, new drugs, such as gliflozines, these ones so far tested in a diabetic setting, exert a positive impact on repair processes, limiting HF and its progression.^11^, ^12^ However, cellular and molecular mechanisms that confer protection from the development of HF in diabetes with gliflozines are largely unknown. In this context, we hypothesized that a specific gliflozine, empagliflozin, can specifically tackle the processes of CSC and cardiac senescence that ultimately favour HF. We then achieved this by comparing a condition of accelerated senescence—diabetes—vs ‘normal’ controls and investigated the mechanism of action of empagliflozin compared to untreated diabetic conditions in a murine model. We subsequently measured senescence markers in vitro and in vivo in the murine model of streptozotocin‐induced diabetes and aimed at interfering with such pathways by empagliflozin.

## 2 METHODS

### 2.1 Materials

d‐glucose and d‐mannitol (this latter devoid of metabolic activities and used as a purely hyperosmolar controls) were purchased from Sigma. Empagliflozin was purchased from Boehringer Ingelheim.

### 2.2 Isolation of cardiospheres and cardiac stromal cells

The murine tissue was derived from hearts (6 months‐old adult mice) of male C57BL/6 mice (Charles River Italia). The isolated myocardial tissue was cut into 1 mm pieces, washed with Ca‐Mg‐free phosphate‐buffered solution (PBS) (Invitrogen) and digested for 5 minutes at 37°C with 0.2% trypsin (Invitrogen). The obtained cells were discarded, and the remaining tissue fragments were washed with complete explant medium (Iscove's Modified Dulbecco's Medium [IMDM] supplemented with 10% foetal calf serum, 100 U/mL penicillin G, 100 μg/mL streptomycin, 2 mmol/L l‐glutamine, and 0.1 mmol/L 2‐mercaptoethanol) and cultured as explants in fibronectin‐coated plates in complete explant medium at 37°C and 5% CO_2_. After 3 weeks, a layer of fibroblast‐like cells was generated from adherent explants on which small, phase‐bright cells migrated. These phase‐bright cells were collected by pooling two washes with Ca‐Mg‐free PBS, one wash with 0.53 mmol/L EDTA (Versene, Invitrogen) (1‐2 minutes), and one wash with 0.5 g/L trypsin and 0.53 mmol/L EDTA (Invitrogen) (2‐3 minutes) at room temperature. 4 × 10^5^ cells were seeded in poly‐d‐lysine‐coated 24 multi‐well plates (BD Bioscences) in cardiosphere (CSp) growing medium (35% complete IMDM/65% DMEM‐Ham F‐12 mix with antibiotics and l‐glutamine, as in complete explant medium), containing cadiomyogenic cocktail of growth factors including 2% B27 (containing 3.5 mg/mL human recombinant insulin), 0.1 mmol/L 2 mercaptoethanol, 10 ng/mL epidermal growth factor, 20 ng/mL basic fibroblast growth factor, 40 nmol/L cardiotrophin‐1 and 40 nmol/L thrombin. CSps at passage 1 and CSC were used for the experiments as indicated below.

### 2.3 Cardiospheres and cardiac stromal cell treatments

CSps at passage 1 and subconfluent CSC were synchronized by starvation in IMDM supplemented with 2% foetal calf serum, 100 U/mL penicillin G, 100 μg/mL streptomycin, 2 mmol/L l‐glutamine for 24 hours, then incubated for 3 or 48 hours with 5.5 mmol/L glucose (normoglycaemia, basal), high glucose (12‐5 and 30.5 mmol/L, HG) or a hyperosmolar control (mannitol 7.0 and 25 mmol/L, HM) in the presence or absence of empagliflozin (100 and 500 nmol/L). Cell viability after treatments was assessed by cell morphology and cell count under phase‐contrast microscopy, trypan blue exclusion and total protein content.

### 2.4 Animal care and experimental procedures

Male C57BL/6 mice (body weight: 30 ± 4 g, 6 months old) were purchased from Charles River Italia. The mice were housed under a 12 hour light/dark cycle (7 am‐7 pm) in temperature and humidity controlled rooms and were provided with ad libitum rodent chow (Teklad 7001, 4.4%; Harlan Teklad Global Diets) and water. Type 1 diabetes mellitus (T1DM) was induced by one single intraperitoneal injection of streptozotocin (STZ, Sigma‐Aldrich) at 150 mg/kg dissolved in 0.01 mol/L citrate buffer (pH 4.5).^13^ Control mice received citrate buffer injections (pH 4.5). Blood glucose was tested using a glucometer (Accuchek Nano, Roche) one week after STZ through tail vein blood collection. Diabetes was defined by blood glucose equal to 280.9 ± 35.8 mg/dL after a six‐hour daytime fast. Mice with lower levels were excluded from the study. Empagliflozin was dissolved in water and administered to mice in the experimental group (30 mg/kg) by oral gavage for 1 month, whereas the vehicle group was given the same volume of water. For echocardiograms and minor procedures, the mice were anaesthetized by intraperitoneal injection of ketamine (100 mg/kg, Clorketam; Vétoquinol). The animals were then killed under 2% isoflurane anaesthesia by cardiac puncture (Online Supplement, Online Figure 1). All procedures were approved by the local Institutional Ethics Committee for Animal Research (Protocol number 176/2019‐R released in February 25 2019). All studies conformed to the Guidelines from Directive 2010/63 EU of the European Parliament on the protection of animals used for scientific purpose of the NIH guidelines.

### 2.5 Echocardiography

Using a portable ultrasound apparatus (Esaote) equipped with a 21‐MHz linear probe, we performed trans‐thoracic echocardiography 1 month after treatments to assess the functional effects of each treatment. The mice were anaesthetized (ketamine, 100 mg/kg) and placed in the left lateral decubitus position. Two‐dimensional (B‐mode) and mono‐dimensional mode (M‐mode) images of parasternal short‐axis and long‐axis views were acquired to position the Motion‐mode (M‐mode) cursor at the level of the papillary muscles and perpendicular to the interventricular septum and left ventricular (LV) free‐wall. To evaluate LV structural changes, several parameters from M‐mode were measured (ie LV end‐diastolic diameter [LVEDD] and LV end‐systolic diameter [LVESD]). Left ventricular fractional shortening (FS%) was calculated as: FS (%) = ([LVEDD − LVESD)/LVEDD] × 100. Left ventricular ejection fraction (EF%) was calculated as an index of systolic function: EF (%) = ([LVEDD_3_ − LVESD_3_)/LVEDD_3_] × 100. The same parameters were measured on control, non‐manipulated healthy animals. Each measurement was obtained by averaging the results of 3 consecutive heartbeats. Individuals conducting the echocardiography were blinded to the animal treatment groups.

### 2.6 Tissue preparation and histological evaluations

After induction of anaesthesia, the hearts of diabetic and age/sex‐matched C57/BL6 control mice were removed and embedded in OCT without fixation or in paraffin after formalin fixation, respectively. The blocks were transversely cut to obtain 15 sections (each 5 μm thick): 5 from the base of the ventricles, 5 from the middle of the ventricles and 5 from the apex. All histological sections were analysed in a blinded fashion. Fibrotic areas were analysed on the paraffin‐embedded sections by picro‐sirius staining. Colour digital images were obtained, and the extent of LV fibrosis was evaluated with the ImageJ software (NIH, http://rsb.info.nih.gov/ij) in five randomly chosen cardiac areas per each heart section and expressed as % of the total LV area.

SA‐b‐gal activity was detected on frozen myocardial samples by the SA‐b‐gal Activity Senescence‐Associated‐β‐Galactosidase Staining (Cell Biolabs, Inc), as previously described.^14^ The frozen sections were post‐fixed in 4% paraformaldehyde at room temperature for 15 minutes, rinsed with sterile PBS and incubated overnight with fresh senescence‐associated β‐galactosidase staining solution (1 mg/mL X‐gal in 40 mmol/L citric acid/sodium phosphate, pH 6.0, 5 mmol/L potassium ferricyanide, 5 mmol/L potassium ferrocyanide, 150 mmol/L sodium chloride, 2 mmol/L magnesium chloride) at 37°C. Then, the staining solution was removed, the sections were immersed in 70% glycerol, and the development of the blue colour was assessed under standard light microscope. The extent of the blue‐stained area was evaluated with the ImageJ software (NIH, http://rsb.info.nih.gov/ij) in five randomly chosen cardiac area per each heart section and expressed as % of total LV area.

### 2.7 Detection of senescent cells in CSC cultures

Senescent cells in CSC cultures were detected by SA‐β‐gal Activity Senescence‐Associated‐β‐Galactosidase Staining (Cell Biolabs, Inc), as previously described.^14^ Briefly, CSC were plated at 2 × 10^5^ cells per well in a 6‐well dish, grown overnight and then incubated for 48 hours with 5.5 mmol/L glucose (normoglycaemia, basal), HG (12‐5 and 30.5 mmol/L, HG) or a hyperosmolar control (mannitol 7.0 and 25 mmol/L, HM) in the presence or absence of empagliflozin (100 nmol/L). The next day, cells were rinsed with sterile PBS, fixed with 0.5% glutaraldehyde at room temperature for 15 minutes and then rinsed with sterile PBS. The cells were stained overnight with fresh senescence‐associated β‐galactosidase staining solution at 37°C. Then, the staining solution was removed and the cells were stored in PBS. Senescent cells showed marked perinuclear blue staining and non‐senescent cells did not exhibit this stain. A standard light microscope was used to count the number of senescence‐associated β‐galactosidase positive cells and the total number of cells over 5 microscope fields per sample. The percentage of senescence was calculated by dividing the average number of senescence‐associated β‐galactosidase positive cells by the average number of total cells and multiplying by 100.

### 2.8 Immunoblotting

Total protein extracts of the whole left cardiac ventricle from the mice or CSC in each experimental group were isolated in an ice‐cold RadioImmuno Precipitation Assay. The proteins were separated under reducing conditions and electroblotted onto polyvinylidene fluoride membrane (Immobilon‐P, Millipore). After blocking, the membranes with proteins harvested from CSC were incubated overnight at 4°C with the following primary antibodies: (a) insulin receptor‐α subunit (IRα, Santa Cruz Biotechnology), (b) insulin receptor substrate type 1 (IRS‐1, Santa Cruz), (c) phosphorylated isoform of Akt (Cell Signaling), (d) phosphorylated isoform of p38 (Santa Cruz), as previously described.^15^ The membranes with proteins harvested from the hearts were incubated overnight at 4°C with primary antibody against type III collagen (Sigma‐Aldrich, 1:10 000 dilution). Equal loading/equal protein transfer was verified by stripping and reprobing the blot with anti‐beta actin (Sigma).

### 2.9 Statistical analysis

Two‐group comparisons were performed by Student's *t* test for unpaired values. Comparisons of three groups or more were performed by analysis of variance (ANOVA), and the existence of individual differences, in case of significant F‐values at ANOVA, tested by Scheffé’s multiple contrasts.

## 3 RESULTS

### 3.1 Empagliflozin increases the number of cardiospheres and reduces the senescence of cardiac stromal cells in high glucose culture conditions

**Figure 1:**
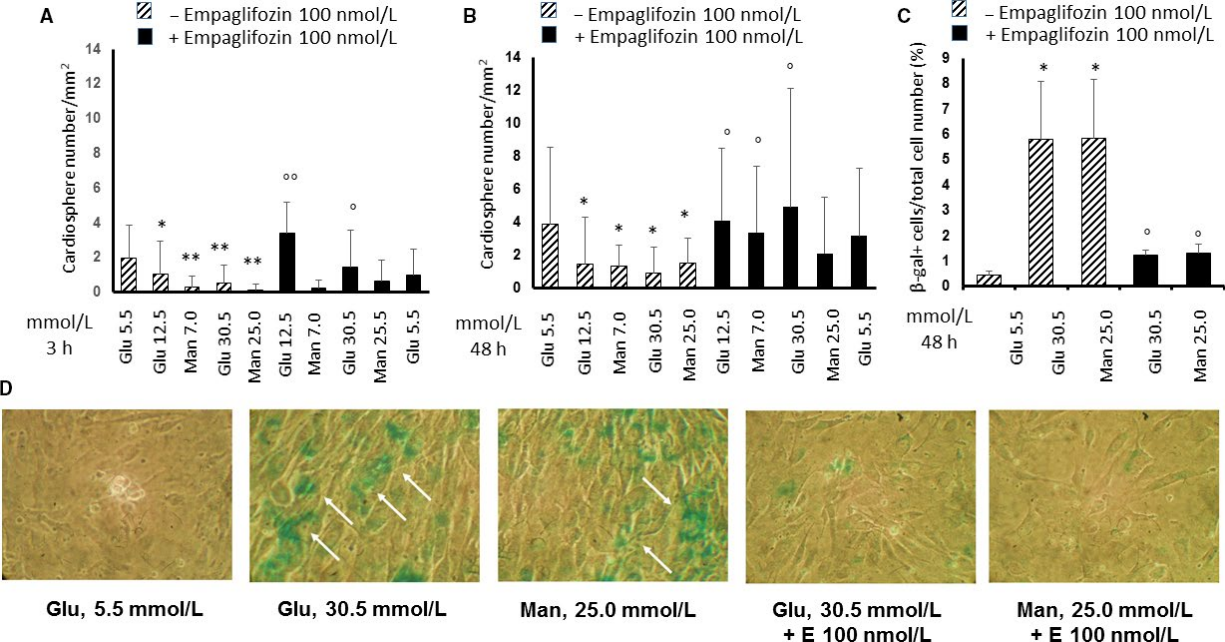
Effects of high glucose and high mannitol with or without empagliflozin on cardiosphere number and senescence of murine cardiac stromal cells. Panels A and B, Quantitation of murine cardiospheres in multiple microscope fields, treated with normal glucose (5.5 mmol/L) or high glucose (12.5 and 30 mmol/L) or high mannitol (7.0 and 25 mmol/L) for 3 h (panel A) and 48 h (Panel B), in the presence or absence of 100 nmol/L empagliflozin. The graph bars showing the total number of cardiospheres over 5 microscope fields per sample represent means ± standard deviations (n = 3 independent experiments). Panel C, Quantitation of senescent cells in multiple microscope fields from murine cardiac stromal cells (CSC) treated with low (5.5 mmol/L) or high glucose (30 mmol/L) or high mannitol (25 mmol/L) for 48 h, in the presence or absence of 100 nmol/L empagliflozin. The graph bars, showing the ratio between the number of senescence‐associated β‐galactosidase (SA‐βGal) positive cells and the total number of cells over 5 microscope fields per sample, represent means ± standard deviations (n = 3 independent experiments). Panel D, Representative SA‐βGal staining images of CSC treated with normal glucose (5.5 mmol/L) or high glucose (30 mmol/L) or high mannitol (25 mmol/L) for 48 h, in the presence or absence of 100 nmol/L empagliflozin, as indicated by the blue staining (arrows). All images are shown at 10× magnification. *P < .05 and **P < .01 vs. normal glucose; ^°^ *P* < .05 and ^°°^ *P* < .01 vs without empagliflozin. E, empagliflozin; Glu, glucose; Man, mannitol; SA‐βGal, senescence‐associated β‐galactosidase

HG (12.5 and 30 mmol/L) and its hyperosmolar control mannitol (7.0 and 25 mmol/L) reduced the number of CSps compared to 5.5 mmol/L glucose at 3 (Figure 1A) and 48 hours after treatment (Figure 1B). HG (30 mmol/L) and HM (25 mmol/L) increased the number of SAβ‐Gal‐positive CSC compared to 5.5 mmol/L glucose at 48 hours after treatment (Figure 1C,D). These results indicate that HG interferes with CSps formation and exerts a pro‐senescent effect on CSC in part through a hyperosmolar mechanism. These effects were significantly attenuated by 100 nmol/L empagliflozin targeting SGLT‐2 (Figure 1A‐D). Under basal conditions in the absence of HG and HM, empagliflozin 100 nmol/L did not show any effect on the cardiosphere number (Figure 1A,B). In contrast, 500 nmol/L empagliflozin exerted cytotoxic effects on CSps by decreasing their number at 3 and 48 hours after treatment (data not shown).

### 3.2 Empagliflozin increases the expression of phosphorylated Akt, while decreases the expression of phosphorylated p38 in cardiac stromal cells under high glucose culture conditions

**Figure 2:**
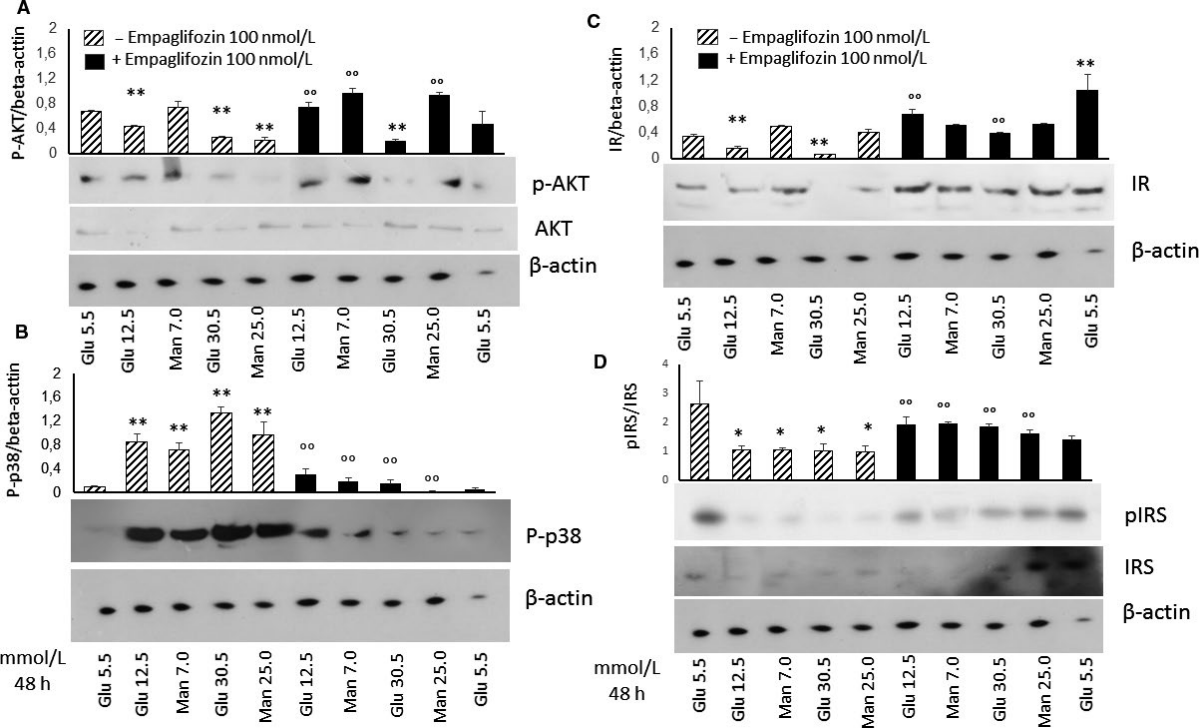
Effects of high glucose and high mannitol with or without empagliflozin on the expression of pAkt, P‐P38, IR‐1α and IRS‐1 in murine cardiac stromal cells. Western analysis of the pro‐survival marker pAkt and AKT (A), pro‐inflammatory marker P‐P38 (B), IR‐1α (C), IRS‐1 and pIRS‐1 (D) in murine cardiac stromal cells treated with normal glucose (5.5 mmol/L) or high glucose (12.5 and 30 mmol/L) or high mannitol (7.0 and 25 mmol/L) for 48 h, in the presence or absence of 100 nmol/L empagliflozin, with β‐actin serving as a loading control. The graph bars represent means ± standard deviations (n = 3 independent experiments). **P < .01 vs normal glucose; ^°°^ *P* < .01 vs without empagliflozin. Glu, glucose; IR, insulin receptor; IRS, Insulin receptor substrate; Man, mannitol; pAkt, phosphorilated Akt; P‐p38, phosphorylated p38

PI3K/Akt/eNOS is one of two parallel pathways activated by insulin after binding to its receptor.^16^ In particular, Akt is an important signalling molecule involved in the regulation of cell survival.^17^ We have recently shown that HG impairs the PI3K/Akt/eNOS pathway and shifts insulin signalling towards the activation of mitogenic and pro‐inflammatory effectors, such as p38 and ERK1/2.^18^ Here, we investigated the effects of HG and HM in phosphorylation of the pro‐survival marker Akt and the pro‐inflammatory marker p38 in CSC. In HG and HM, the levels of Ser473‐phosphorylated Akt in CSC were reduced (Figure 2A). This was accompanied by an increase in the levels of the pro‐inflammatory marker phosphorylated p38 (Figure 2B), indicating a selective down‐regulation of the PI3/Akt pathway caused by HG through a hyperosmolar mechanism, which spares the mitogenic insulin signalling pathway. These effects were significantly attenuated by 100 nmol/L empagliflozin (Figure 2A,B). Under basal conditions in the absence of HG and HM, empagliflozin 100 nmol/ L did not show any effect on the expression of AKT nor P‐p38 (Figure 2A,B). To examine whether the PI3K/Akt down‐regulation upon incubation with HG and HM is due to insulin resistance through insulin receptor (IR)‐α degradation, we semi‐quantified the total amount of IR‐α by immunoblotting. The expression of IR‐α was reduced by HG but was not influenced by HM (Figure 2C). 100 nmol/L empaglifozin was able to restore IR expression in CSC under HG culture conditions and induced it under in normoglycaemia, suggesting an insulin‐sensitizing effect both in normoglycaemic and hyperglycaemic conditions (Figure 2C). We also tested the possibility that incubation with HG and HM could affect the protein levels of phosphorylated (pIRS)‐1 and total insulin receptor substrate (IRS)‐1. Unlike IR‐α, incubation with full range concentrations of HM and HG reduced pIRS‐1 and IRS‐1 (Figure 2D), indicating that a reduction in insulin signalling by HG‐induced hyperosmolar stress should depend by a disturbance in the signalling pathway downstream of the IR. 100 nmol/L empaglifozin was able to restore the expression of IRS‐1 in the CSC under HG and HM culture conditions, suggesting an insulin‐sensitizing effect (Figure 2D).

### 3.3 Empagliflozin reduced high glucose‐induced cardiac dysfunction in streptozotocin‐treated mice

**Figure 3:**
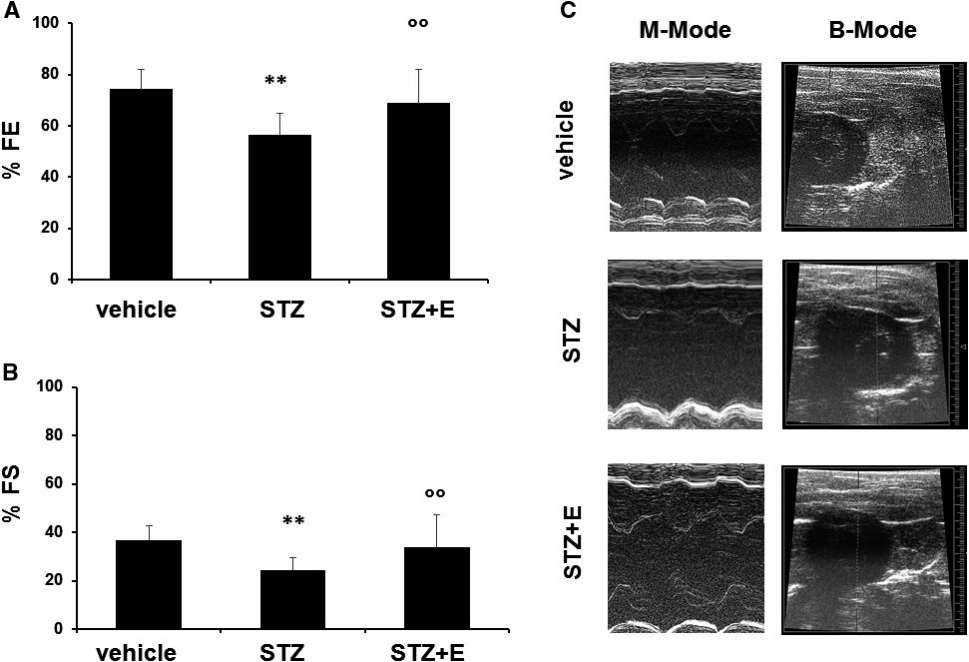
Effects of streptozotocin and empagliflozin on cardiac function in mice. Panels A, Ejection Fraction (EF) and B, Fractional Shortening (FS) measured by echocardiography in the different treatment groups such as control (vehicle), streptozotocin‐treated mice and streptozotocin + empagliflozin‐treated mice. Panels C, Representative M‐mode and B‐mode images recorded by echocardiography in the different treatment groups. Data are expressed as means ± standard deviations (n = 5 mice per treatment group). **P < .01 vs vehicle‐treated mice; ^°°^ *P* < .01 vs without empagliflozin. E, empagliflozin; STZ, streptozotocin

To study the impact of empagliflozin on systolic function of the left ventricle, trans‐thoracic echocardiography was performed in mice treated with STZ, or STZ + emapgliflozin or vehicle (saline). Figure 3 shows the representative images of the echocardiographic analyses. Left ventricular ejection fraction (LVEF), Left ventricular fractional short circuit (LVFS), left ventricular diastolic end (LVEDD) and left ventricular systolic end (LVESD) diameters were assessed to determine left ventricular systolic function. Compared to the vehicle, the administration of STZ induced a significant reduction of systolic function as represented by lower LVEF and LVFS (Figure 3). Empagliflozin significantly attenuated these changes (Figure 3).

### 3.4 Empagliflozin reduced high glucose—induced cardiac fibrosis and senescence

**Figure 4:**
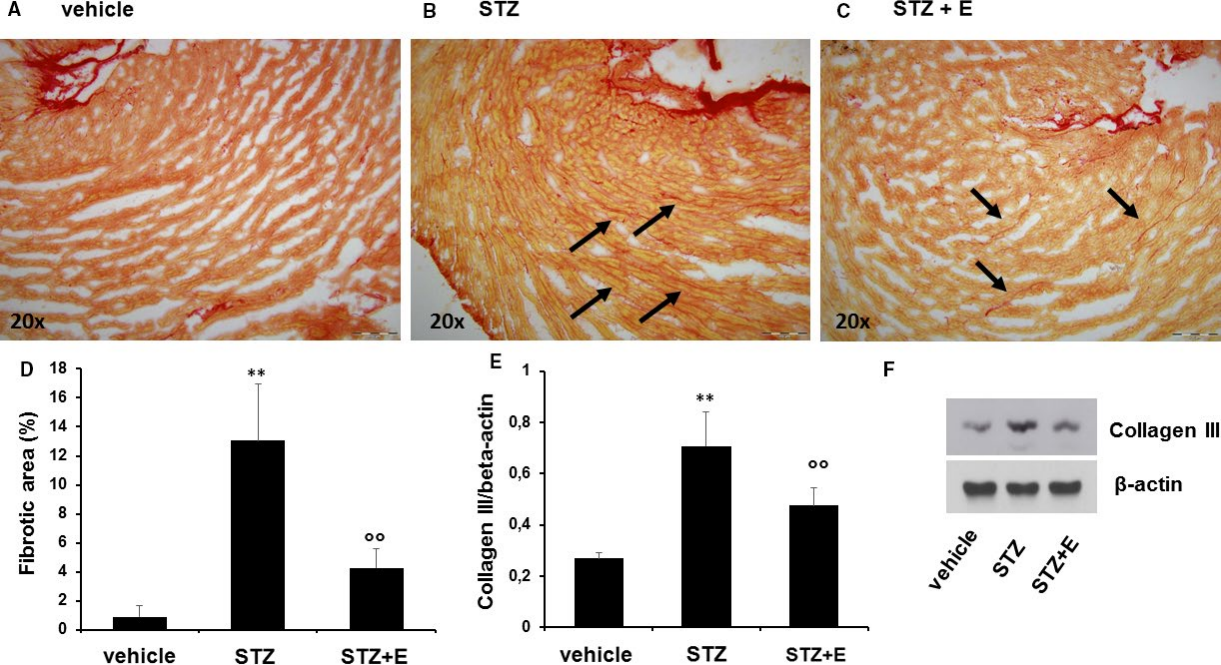
Effects of streptozotocin and empagliflozin on cardiac fibrosis in mice. Panels A‐C, Picro‐sirius red staining showing the collagen content in left ventricle sections from control (vehicle), streptozotocin‐treated mice and streptozotocin + empagliflozin‐treated mice. All images are shown at 20× original magnification. Panel D, Collagen content quantified on picro‐sirius red staining using the ImageJ software. Panel E, Quantification of collagen type III expression by western analysis. Panel F, Representative western blot showing type III collagen expression in each treatment group. β‐actin levels were assessed as a loading control. Data are expressed as means ± standard deviation (n = 5 mice per treatment group). **P < .01 vs vehicle‐treated mice; ^°°^ *P* < .01 vs without empagliflozin. E, empagliflozin; STZ, streptozotocin

**Figure 5:**
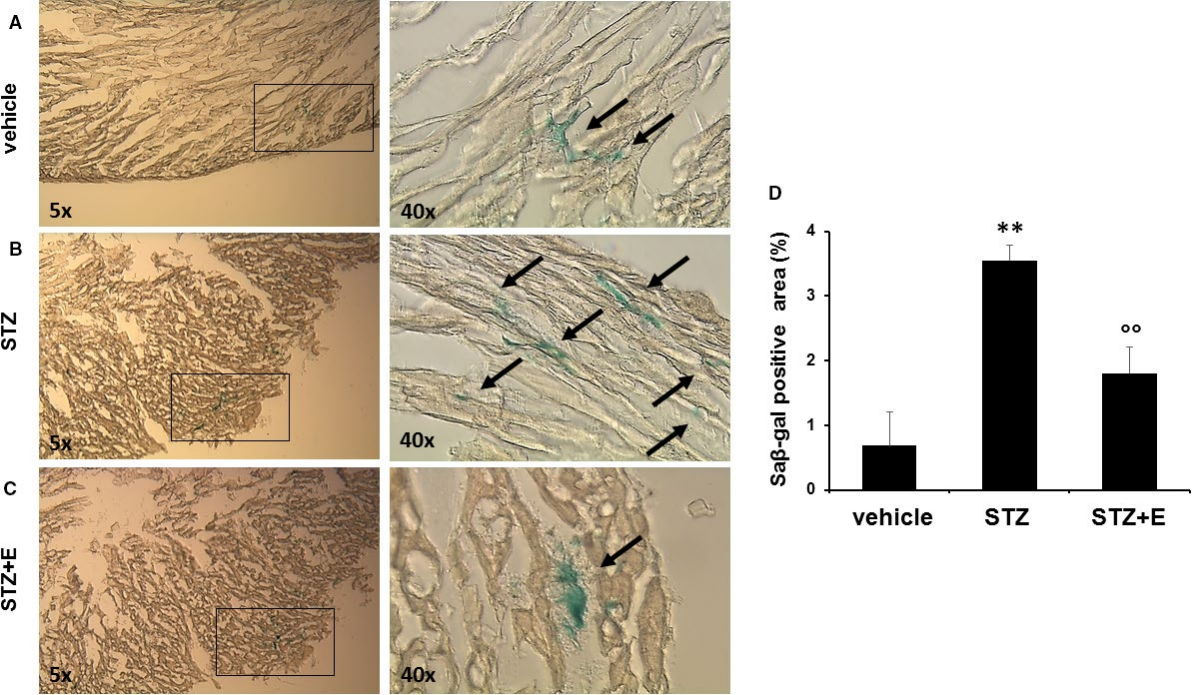
Effects of streptozotocin and empagliflozin on cardiac senescence‐associated β‐galactosidase activity in mice. Panels A‐C, Senescence β‐galactosidase (SA β‐Gal) staining (magnification 5× and 40×) and quantitative evaluations (Panel D) in cardiac tissue. Data are expressed as means ± standard deviations (n = 5 mice per treatment group). **P < .01 vs vehicle‐treated mice; ^°°^ *P* < .01 vs without empagliflozin. E, empagliflozin; STZ, streptozotocin

To study the effect of empagliflozin on HG‐induced cardiac fibrosis, we performed histopathological staining and western blotting analysis. The Sirius red staining showed that the fibrotic area was increased in STZ‐treated mice compared to the vehicle (Figure 4A,B). The collagen area was smaller in the STZ + empagliflozin treatment group than in the STZ treatment group (Figure 4C). Western blotting showed that STZ treatment increased the expression of the fibrosis marker collagen III (Figure 4D). Notably, the expression of collagen type III was significantly reduced upon empagliflozin treatment (Figure 4E,F). STZ induced a significantly higher percentage of SA‐β‐gal‐positive cardiac area, which was reduced by empagliflozin treatment (Figure 5).

## 4 DISCUSSION

**Figure 6:**
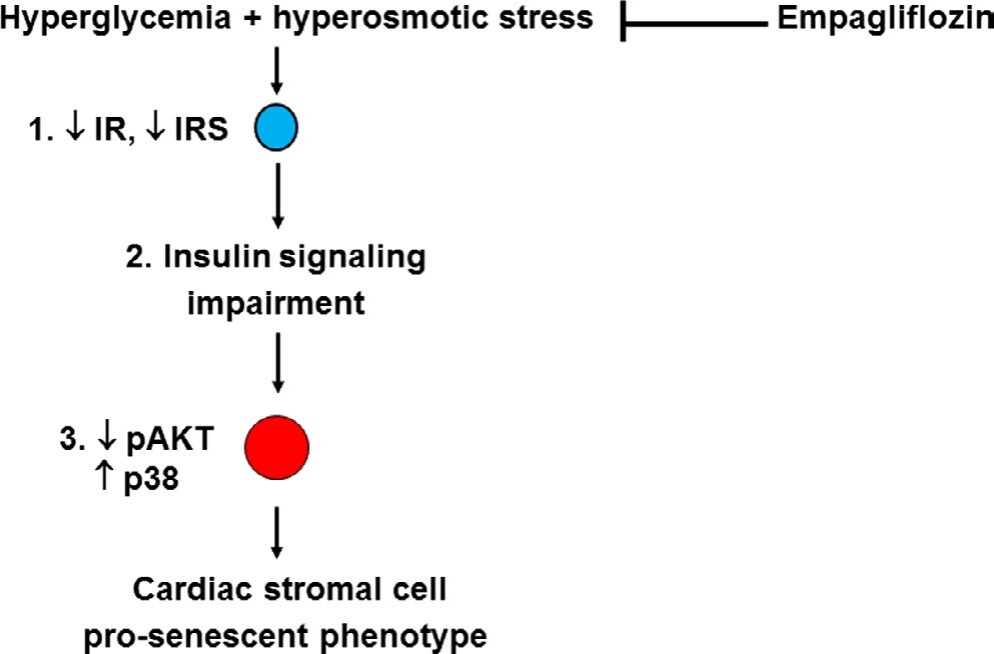
A scheme of the putative signalling pathway through which empagliflozin target cardiac stromal cell senescence induced by hyperglycaemia and hyperosmotic, based on findings from the in vitro part of the present study. In the plasma membrane, hyperglycaemia induces hyperosmotic stress, and together, these metabolic and biophysical stressors impair the ability of cardiac stromal cells to respond to insulin. This causes the perturbation of the pro‐survival PI3K/Akt and pro‐inflammatory p38 pathways with insulin preserving its signalling capacity through the mitogenic and pro‐inflammatory arm, leading to the development and progression of a pro‐senescent phenotype. Empagliflozin is able to revert CSC senescence by targeting the insulin signalling pathway

In the present study, we demonstrated that HG, mimicking the in vivo conditions of type 1 and type 2 diabetes, induces cellular senescence in CSC. This effect is accompanied by the attenuation of the anti‐inflammatory and pro‐survival insulin signalling in CSC through a down‐regulation of the PI3K/Akt pathway. The effects of HG on the PI3K/Akt pathway depend in part on the hyperosmolar component of HG, since they are mimicked by CSC exposure to the metabolically inactive monosaccharide mannitol. Since these effects are observed only in the PI3K/Akt pathway, while in the same conditions CSC do not lose the ability to respond to insulin with the activation of P38, these data support the hypothesis that HG promotes an imbalance in CSC insulin signalling between pro‐survival and pro‐inflammatory arms, with insulin preserving its signalling capacity through the mitogenic and pro‐inflammatory arm. Attenuated insulin signalling can also result from changes in insulin resistance and/or post‐receptor signalling.^18^ We show here that exposure to HG affects the total amount of IR‐α and IRS‐1 levels, with the hyperosmolar component starting to act on the signal further downstream of the IRS‐1. Overall, this indicates that the altered CSC responsiveness to insulin is due to a disturbance in the signalling pathway starting from IR‐α (Figure 6).

Hyperglycaemia has been reported to contribute to tissue injury, particularly in heart disease.^19^, ^20^ Several signalling pathways have been analysed and targeted in cardiac fibrosis, senescence and ischaemia, on a dysmetabolic or cardiotoxic basis, including activation of the Stat3‐mediated pathway.^21^, ^22^, ^23^ In non‐diabetic mice undergoing ischaemia‐reperfusion injury, empagliflozin reduced myocardial infart size through STAT‐3.^24^ Guo et al^20^ reported that HG caused cell senescence in H9C2 and foetal myoblasts through oxidative stress and nuclear factor‐κB (NF‐κB)‐mediated inflammation. Mechanisms through which HG promotes cell senescence are probabily multiple.^25^ In the present study, we demonstrated an association between down‐regulation of the PI3K/Akt signalling pathway and induction of senescence in CSC exposed to HG. Akt regulates the expression and activity of several targets involved in apoptosis and proliferation. For example, Akt induces an up‐regulation of cyclin D1 which is responsible for the transition through G1/S phases^26^, ^27^, ^28^, ^29^, ^30^ Moreover, Akt is known to phosphorylate and inactivate proapoptotic factors, such as BCL2‐antagonist of cell death (BAD)^31^ and procaspase‐9,^32^ thus promoting cell survival. Importantly, the present study revealed the importance of empagliflozin on HG‐induced cell senescence and the PI3K/Akt down‐regulation. We have here provided new data showing the direct effects of empagliflozin in vitro on the murine CSC phenotype by attenuation of their pro‐senescent phenotype. In fact, empagliflozin clearly suppressed the HG‐induced cell senescence and reverted the PI3K/Akt down‐regulation in cultured CSC, suggesting a pro‐survival effect of the drug. Furthermore, we here provide data showing the antifibrotic and anti‐senescent effects of empagliflozin in vivo in the murine heart. These findings are consistent with recent animal studies showing that empagliflozin reduces myocardial interstitial fibrosis and remodelling in hypertensive rats.^33^, ^34^, ^35^ Although the SGLT1 isoform is the only form expressed in the capillaries and the myocardium of human and rodent hearts,^36^ and we did not find any consistent data supporting the expression of SGLT2 receptor in murine CSC and in the heart, it is possible that empagliflozin has off‐target effects outside of SGLT2‐mediated pathways in the heart, regulating CSC activity. CSC are a mixed population of non‐cardiomyocyte cells including fibroblasts with paracrine activity, and can therefore represent the cellular target of empagliflozin for its antifibrotic and anti‐senescence effects. Indeed, one of the relevant component of CSC, the cardiac fibroblasts, is known to express sodium‐hydrogen exchanger 1 (NHE‐1),^37^, ^38^ which is an established molecular target of SGLT2 inhibitors, including empagliflozin.^39^ We have previously shown that NHE‐1 is one of the molecular targets of hyperosmolar stress, capable of mediating the down‐regulation of the PI3/Akt/eNOS signalling pathway induced by hyperosmolar stress in endothelial cells exposed to HG and HM, and that this effect is reverted by NHE‐1 blockade through cariporide.^40^, ^41^, ^42^ In the present work, we have demonstrated a similar down‐regulation of the PI3/Akt signalling pathway exerted by hyperosmolar stress in CSCs exposed to HG and HM, and that this effect is reversed by empagliflozin. Inhibition of NHE‐1 attenuated cardiac fibrosis and HF in rat models of high blood pressure,^43^, ^44^ and cariporide treatment caused a significant increase in the lifespan of both hypertensive and normotensive rats, attenuating cardiac fibrosis typically associated with ageing.^45^

We recognize limitations of this study. A first one is that we here used murine cardiac samples to explain effects of empagliflozin in humans. Indeed, there are intrinsic constraints when using murine specimens, including diverse pharmacodynamic and pharmacokinetic profiles of the drug. Another limitation is that the different mechanisms by which Akt can exert a pro‐survival action have not been studied in our cell system of CSC exposed to HG, and this needs to be studied in the future. Finally, this study does not provide direct data to support the mechanistic hypothesis of the antifibrotic and anti‐senescence effects of empagliflozin on the heart.

In conclusion, our study shows that HG‐induced senescence and cardiac fibrosis are reduced by empagliflozin possibly through SGLT2 off‐target effects. These effects may help explain unexpected benefits of empagliflozin on cardiac function in patients with diabetes.

## CONFLICT OF INTEREST

The authors declare no conflict of interest.

## AUTHOR CONTRIBUTION

**Rosalinda Madonna:** Conceptualization (equal); Data curation (equal); Formal analysis (equal); Funding acquisition (equal); Investigation (equal); Methodology (equal); Supervision (equal); Writing‐original draft (equal). **Vanessa Doria:** Data curation (supporting); Methodology (supporting). **Ilaria Minnucci:** Data curation (supporting); Methodology (supporting). **Angela Pucci:** Data curation (supporting); Formal analysis (supporting); Methodology (supporting); Writing‐original draft (supporting). **Donato Sante PIerdomenico:** Formal analysis (equal); Methodology (supporting); Resources (supporting); Validation (equal); Writing‐original draft (supporting). **Raffaele De Caterina:** Conceptualization (lead); Data curation (supporting); Funding acquisition (lead); Methodology (supporting); Project administration (lead); Resources (lead); Supervision (lead); Validation (lead); Writing‐review & editing (lead).

## Supporting information

Online Figure

Online Supplement

## Data Availability

Data available on request from the authors.

## References

[1] Timmis A , Townsend N , Gale C , et al. European society of cardiology: cardiovascular disease statistics 2017. Eur Heart J. 2018;39:508‐579. 29190377 10.1093/eurheartj/ehx628

[2] Xin M , Olson EN , Bassel‐Duby R . Mending broken hearts: cardiac development as a basis for adult heart regeneration and repair. Nat Rev Mol Cell Biol. 2013;14:529‐541. 23839576 10.1038/nrm3619 PMC3757945

[3] Olson EN . A decade of discoveries in cardiac biology. Nat Med. 2004;10:467‐474. 15122248 10.1038/nm0504-467

[4] Mayourian J , Ceholski DK , Gonzalez DM , et al. Physiologic, pathologic, and therapeutic paracrine modulation of cardiac excitation‐contraction coupling. Circ Res. 2018;122:167‐183. 29301848 10.1161/CIRCRESAHA.117.311589 PMC5886757

[5] Shen D , Shen M , Liang H , et al. Therapeutic benefits of CD90‐negative cardiac stromal cells in rats with a 30‐day chronic infarct. J Cell Mol Med. 2018;22:1984‐1991. 29341439 10.1111/jcmm.13517 PMC5824400

[6] Forbes SJ , Rosenthal N . Preparing the ground for tissue regeneration: from mechanism to therapy. Nat Med. 2014;20:857‐869. 25100531 10.1038/nm.3653

[7] Cencioni C , Atlante S , Savoia M , et al. The double life of cardiac mesenchymal cells: epimetabolic sensors and therapeutic assets for heart regeneration. Pharmacol Ther. 2017;171:43‐55. 27742569 10.1016/j.pharmthera.2016.10.005

[8] Perkisas S , Vandewoude M . Where frailty meets diabetes. Diabetes Metab Res Rev. 2016;32(Suppl 1):261‐267. 26453435 10.1002/dmrr.2743

[9] Morley JE . Diabetes and aging: epidemiologic overview. Clin Geriatr Med. 2008;24:395‐405. 18672179 10.1016/j.cger.2008.03.005

[10] Moore‐Morris T , Guimarães‐Camboa N , Yutzey K , Pucéat M , Evans S . Cardiac fibroblasts: from development to heart failure. J Mol Med. 2015;93:823‐830. 26169532 10.1007/s00109-015-1314-y PMC4512919

[11] Persson F , Nystrom T , Jorgensen ME , et al. Dapagliflozin is associated with lower risk of cardiovascular events and all‐cause mortality in people with type 2 diabetes (CVD‐REAL Nordic) when compared with dipeptidyl peptidase‐4 inhibitor therapy: a multinational observational study. Diabetes Obes Metab. 2018;20:344‐351. 28771923 10.1111/dom.13077 PMC5811811

[12] Zinman B , Wanner C , Lachin JM , et al. Empagliflozin, cardiovascular outcomes, and mortality in type 2 diabetes. N Engl J Med. 2015;373:2117‐2128. 26378978 10.1056/NEJMoa1504720

[13] Wu KK , Huan Y . Streptozotocin‐induced diabetic models in mice and rats. Curr Protoc Pharmacol. 2008;Chapter 5:Unit 5 47. 10.1002/0471141755.ph0547s40 22294227

[14] Madonna R , Pieragostino D , Cufaro MC , et al. Ponatinib induces vascular toxicity through the notch‐1 signaling pathway. J Clin Med. 2020;9(3):820. 10.3390/jcm9030820 PMC7141219 32197359

[15] Madonna R , Di Napoli P , Massaro M , et al. Simvastatin attenuates expression of cytokine‐inducible nitric‐oxide synthase in embryonic cardiac myoblasts. J Biol Chem. 2005;280:13503‐13511. 15705589 10.1074/jbc.M411859200

[16] Nystrom F , Quon M . Insulin signalling: metabolic pathways and mechanisms for specificity. Cell Signal. 1999;11:563‐574. 10433517 10.1016/s0898-6568(99)00025-x

[17] Lee C‐H , Shieh Y‐S , Hsiao F‐C , et al. High glucose induces human endothelial dysfunction through an Axl‐dependent mechanism. Cardiovasc Diabetol. 2014;13:53. 24572151 10.1186/1475-2840-13-53 PMC3941696

[18] Madonna R , Pieragostino D , Rossi C , et al. Simulated hyperglycemia impairs insulin signaling in endothelial cells through a hyperosmolar mechanism. Vascul Pharmacol. 2020;130:106678. 32229255 10.1016/j.vph.2020.106678

[19] Liao Y , Takashima S , Zhao H , et al. Control of plasma glucose with alpha‐glucosidase inhibitor attenuates oxidative stress and slows the progression of heart failure in mice. Cardiovasc Res. 2006;70:107‐116. 16510136 10.1016/j.cardiores.2006.01.021

[20] Guo Y , Zhuang X , Huang Z , et al. Klotho protects the heart from hyperglycemia‐induced injury by inactivating ROS and NF‐κB‐mediated inflammation both in vitro and in vivo. Biochim Biophys Acta Mol Basis Dis. 2018;1864:238‐251. 28982613 10.1016/j.bbadis.2017.09.029

[21] Andreadou I , Efentakis P , Balafas E , et al. Empagliflozin limits myocardial infarction in vivo and cell death in vitro: role of STAT3, mitochondria, and redox aspects. Front Physiol. 2017;8:1077. 29311992 10.3389/fphys.2017.01077 PMC5742117

[22] Penna C , Andreadou I , Aragno M , et al. Effect of hyperglycaemia and diabetes on acute myocardial ischaemia‐reperfusion injury and cardioprotection by ischaemic conditioning protocols. Br J Pharmacol. 2020. 10.1111/bph.14993 PMC7680002 31985828

[23] Penna C , Perrelli M‐G , Tullio F , et al. Diazoxide postconditioning induces mitochondrial protein S‐nitrosylation and a redox‐sensitive mitochondrial phosphorylation/translocation of RISK elements: no role for SAFE. Basic Res Cardiol. 2013;108:371. 23872876 10.1007/s00395-013-0371-z

[24] Nikolaou PE , Efentakis P , Qourah FA , et al. Chronic Empaglifozin treatment reduces myocardial infarct size in non‐diabetic mice through STAT‐3 mediated protection on microvascular endothelial cells and reduction of oxidative stress. Antioxid Redox Signal. 2020. 10.1089/ars.2019.7923 32295413

[25] Madonna R , Novo G , Balistreri C . Cellular and molecular basis of the imbalance between vascular damage and repair in ageing and age‐related diseases: as biomarkers and targets for new treatments. Mech Ageing Dev. 2016;159:22‐30. 26993150 10.1016/j.mad.2016.03.005

[26] Datta S , Brunet A , Greenberg M . Cellular survival: a play in three Akts. Genes Dev. 1999;13:2905‐2927. 10579998 10.1101/gad.13.22.2905

[27] Medema R , Kops G , Bos J , Burgering B . AFX‐like Forkhead transcription factors mediate cell‐cycle regulation by Ras and PKB through p27kip1. Nature. 2000;404:782‐787. 10783894 10.1038/35008115

[28] Shin I , Yakes FM , Rojo F , et al. PKB/Akt mediates cell‐cycle progression by phosphorylation of p27(Kip1) at threonine 157 and modulation of its cellular localization. Nat Med. 2002;8:1145‐1152. 12244301 10.1038/nm759

[29] Viglietto G , Motti ML , Bruni P , et al. Cytoplasmic relocalization and inhibition of the cyclin‐dependent kinase inhibitor p27(Kip1) by PKB/Akt‐mediated phosphorylation in breast cancer. Nat Med. 2002;8:1136‐1144. 12244303 10.1038/nm762

[30] Diehl J , Cheng M , Roussel M , Sherr C . Glycogen synthase kinase‐3beta regulates cyclin D1 proteolysis and subcellular localization. Genes Dev. 1998;12:3499‐3511. 9832503 10.1101/gad.12.22.3499 PMC317244

[31] Datta SR , Dudek H , Tao XU , et al. Akt phosphorylation of BAD couples survival signals to the cell‐intrinsic death machinery. Cell. 1997;91:231‐241. 9346240 10.1016/s0092-8674(00)80405-5

[32] Cardone M , Roy N , Stennicke H , et al. Regulation of cell death protease caspase‐9 by phosphorylation. Science. 1998;282:1318‐1321. 9812896 10.1126/science.282.5392.1318

[33] Kusaka H , Koibuchi N , Hasegawa Y , Ogawa H , Kim‐Mitsuyama S . Empagliflozin lessened cardiac injury and reduced visceral adipocyte hypertrophy in prediabetic rats with metabolic syndrome. Cardiovasc Diabetol. 2016;15:1‐14. 27835975 10.1186/s12933-016-0473-7 PMC5106779

[34] Lin B , Koibuchi N , Hasegawa YU , et al. Glycemic control with empagliflozin, a novel selective SGLT2 inhibitor, ameliorates cardiovascular injury and cognitive dysfunction in obese and type 2 diabetic mice. Cardiovasc Diabetol. 2014;13:1‐15. 25344694 10.1186/s12933-014-0148-1 PMC4219031

[35] Habibi J , Aroor AR , Sowers JR , et al. Sodium glucose transporter 2 (SGLT2) inhibition with empagliflozin improves cardiac diastolic function in a female rodent model of diabetes. Cardiovasc Diabetol. 2017;16:9. 28086951 10.1186/s12933-016-0489-z PMC5237274

[36] Vrhovac I , Balen Eror D , Klessen D , et al. Localizations of Na(+)‐D‐glucose cotransporters SGLT1 and SGLT2 in human kidney and of SGLT1 in human small intestine, liver, lung, and heart. Pflugers Arch. 2015;467:1881‐1898. 25304002 10.1007/s00424-014-1619-7

[37] Maly K , Strese K , Kampfer S . Critical role of protein kinase C a and calcium in growth factor induced activation of the Na�/H� exchanger NHE1. FEBS Lett. 2002;521:205‐210. 12067706 10.1016/s0014-5793(02)02867-3

[38] Orlowski J , Grinstein S . Diversity of the mammalian sodium/proton exchanger SLC9 gene family. Pflugers Arch. 2004;447:549‐565. 12845533 10.1007/s00424-003-1110-3

[39] Uthman L , Baartscheer A , Bleijlevens B , et al. Class effects of SGLT2 inhibitors in mouse cardiomyocytes and hearts: inhibition of Na+/H+ exchanger, lowering of cytosolic Na+ and vasodilation. Diabetologia. 2018;61:722‐726. 29197997 10.1007/s00125-017-4509-7 PMC6448958

[40] Madonna R , Montebello E , Lazzerini G , Zurro M , De Caterina R . NA+/H+ exchanger 1‐ and aquaporin‐1‐dependent hyperosmolarity changes decrease nitric oxide production and induce VCAM‐1 expression in endothelial cells exposed to high glucose. Int J Immunopathol Pharmacol. 2010;23:755‐765. 20943045 10.1177/039463201002300309

[41] Madonna R , De Caterina R . Sodium‐hydrogen exchangers (NHE) in human cardiovascular diseases: interfering strategies and their therapeutic applications. Vascul Pharmacol. 2013;59:127‐130. 24140414 10.1016/j.vph.2013.10.001

[42] Madonna R , De Caterina R . Aquaporin‐1 and sodium‐hydrogen exchangers as pharmacological targets in diabetic atherosclerosis. Curr Drug Targets. 2015;16:361‐365. 25523901 10.2174/1389450116666141219115720

[43] Darmellah A , Baetz D , Prunier F , Tamareille S , Rücker‐Martin C , Feuvray D . Enhanced activity of the myocardial Na+/H+ exchanger contributes to left ventricular hypertrophy in the Goto‐Kakizaki rat model of type 2 diabetes: critical role of Akt. Diabetologia. 2007;50:1335‐1344. 17429605 10.1007/s00125-007-0628-x

[44] Engelhardt S , Hein L , Keller U , Klambt K , Lohse M . Inhibition of Na+/H+ exchange prevents hypertrophy, fibrosis, and heart failure in β1‐adrenergic receptor transgenic mice. Circ Res. 2002;90:814‐819. 11964375 10.1161/01.res.0000014966.97486.c0

[45] Linz W , Busch A . NHE‐1 inhibition: from protection during acute ischaemia/reperfusion to prevention/reversal of myocardial remodeling. Naunyn Schmiedebergs Arch Pharmacol. 2003;368:239‐246. 14504689 10.1007/s00210-003-0808-2

