# Supplementary figures and images for: Empagliflozin reduces the senescence of cardiac stromal cells and improves cardiac function in a murine model of diabetes
Source: J Cell Mol Med. 2020 Sep 17;24(21):12331–40. doi: 10.1111/jcmm.15699 (PMC7687009; doi:10.1111/jcmm.15699)

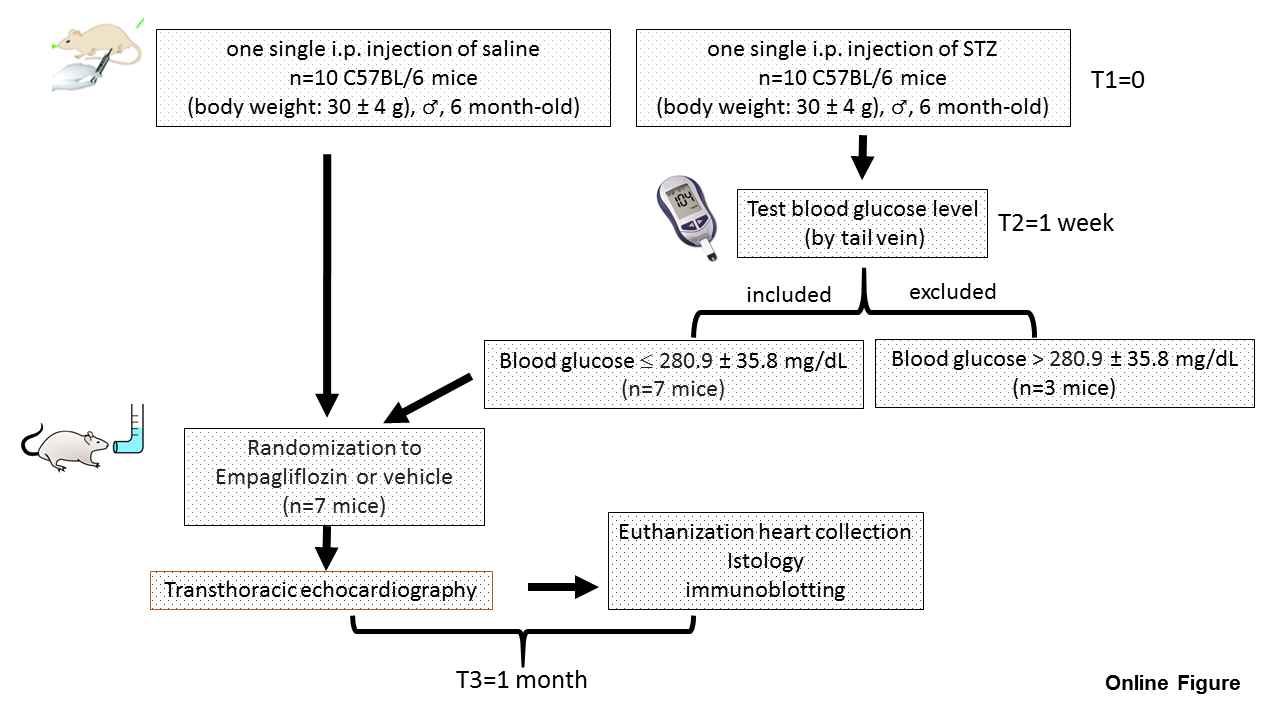

Supplement: Supplementary file 1 — Online Figure [file JCMM-24-12331-s001.TIF]
