# Supplementary material for: Empagliflozin reduces the senescence of cardiac stromal cells and improves cardiac function in a murine model of diabetes
Source: J Cell Mol Med. 2020 Sep 17;24(21):12331–40. doi: 10.1111/jcmm.15699 (PMC7687009; doi:10.1111/jcmm.15699)
Supplement: Supplementary file 2 — Online Supplement [file JCMM-24-12331-s002.docx]

**Empagliflozin reduces the senescence of cardiac stromal cells and improves cardiac function in a murine model of diabetes**

R. Madonna^1^, V. Doria^2^, I. Minnucci^2^, A. Pucci^3^, SD. Pierdomenico^2^*, Raffaele De Caterina^1^*

^1^ Institute of Cardiology, University of Pisa, Pisa, Italy

^2^ Center of Aging Sciences and Translational Medicine - CESI-Met "G. D'Annunzio" University, Chieti-Pescara, Chieti, Italy

^3^ Histopathology Department, Pisa University Hospital, Italy

Running title: Empagliflozin and cardiac senescence

Abstract 238; Main text, including title page, abstract, acknowledgments, references, figures, and figure legends): 5789; Figures: 6; online supplement.

*Correspondence to:

Raffaele De Caterina Sante Donato Pierdomenico

Institute of Cardiology, University of Pisa Institute of Cardiology, University of Chieti

C/o Ospedale Cisanello c/o Ospedale “SS. Annunziata”

Via Paradisa Via dei Vestini

66013 Pisa, Italy 66100 Chieti, Italy

E-mail: [raffaele.decaterina@unipi.it](mailto:raffaele.decaterina@unipi.it) Email: pierdom@unich.it

**Online Supplement**

**Online Figure 1:** Experimental protocol of empagliflozin treatment in the streptozotocin-induced diabetic mice. Legend: i.p., intraperitoneal injection.
